# The Effectiveness of Prophylactic Epinephrine Compared to No Prophylaxis for Postpolypectomy Bleeding in Endoscopic Colorectal Surgery: A Systematic Review and Meta-Analysis

**DOI:** 10.7759/cureus.56778

**Published:** 2024-03-23

**Authors:** Akash Patel, Guy Treves, Isha Samreen, Utsav P Vaghani

**Affiliations:** 1 Internal Medicine, Eisenhower Health, Rancho Mirage, USA; 2 Medicine and Surgery, St. George's University School of Medicine, Irvine, USA; 3 Internal Medicine, Hemet Global Medical Center, Hemet, USA; 4 Internal Medicine, Smt. N.H.L. Municipal Medical College, Ahmedabad, IND

**Keywords:** endoscopic techniques, bleeding prevention, systematic review and meta analysis, prophylactic epinephrine, postpolypectomy bleeding, colonoscopic polypectomy

## Abstract

Colorectal cancer prevention has seen significant advancements with colonoscopic polypectomy, a critical technique in clinical practice. However, postpolypectomy bleeding (PPB), particularly in the resection of large pedunculated polyps, remains a major complication. This systematic review and meta-analysis investigates the efficacy of prophylactic epinephrine injections in preventing PPB, addressing inconsistencies in the literature regarding its effectiveness. Employing a comprehensive search strategy, we rigorously selected studies for inclusion, focusing on those comparing prophylactic epinephrine with no intervention. The risk of bias was assessed using the Cochrane Risk of Bias assessment tool, ensuring a robust and reliable analysis. Our findings, based on an analysis of four studies involving 1,062 patients, indicate a significant reduction in early PPB with epinephrine use, with a marked decrease in bleeding incidence compared to the no-prophylaxis group. However, the impact on delayed bleeding was less conclusive, suggesting the need for further research in this area. Our study thus highlights the effectiveness of epinephrine as a preventive tool in colonoscopic polypectomy while underscoring the complexity of bleeding risks and the necessity for ongoing investigation in optimizing patient outcomes.

## Introduction and background

Colorectal cancer prevention has been significantly advanced by the advent of colonoscopic polypectomy, a technique that has been a mainstay in clinical practice for decades. A major complication of this procedure is postpolypectomy bleeding (PPB), especially prevalent in the resection of large pedunculated polyps due to sizable arterial structures within their stalks. The incidence of PPB varies widely, reported in the literature as ranging from a modest 0.3% to a more concerning 6.1%, with these higher incidences often linked to the removal of larger colonic polyps [1-3].

Various risk factors have been identified as contributing to the likelihood of PPB. These include age-related factors, existing comorbidities, usage of anticoagulant therapies, and specific characteristics of the polyps themselves such as their size and colon location [4-7]. In an effort to mitigate these risks, numerous endoscopic strategies have been explored, among which submucosal injection of epinephrine has emerged as a notable approach. The vasoconstrictive properties of epinephrine are well-established and have demonstrated efficacy in reducing bleeding risks, particularly in cases involving large sessile polyps [8-12].

However, the literature reflects a degree of inconsistency in the outcomes attributed to epinephrine injections, thus underscoring the need for a comprehensive and methodologically robust meta-analysis. Such an analysis is essential to evaluate the effectiveness of epinephrine [13-15].

This systematic review and meta-analysis endeavor to meticulously evaluate the efficacy of prophylactic epinephrine injections in preventing PPB. By offering a comprehensive assessment and synthesizing existing data, our study aims to bridge current research gaps and potentially inform future clinical guidelines, thereby enhancing the standard of care in the field of gastroenterology [16].

## Review

Methods

This systematic review and meta-analysis was meticulously designed to assess the effectiveness of prophylactic epinephrine injections in preventing postpolypectomy bleeding compared to no prophylaxis.

Search Strategy

A detailed and extensive search strategy was implemented, utilizing databases such as PubMed, CINAHL, EBSCO, and Google Scholar. The search was concluded on December 20, 2023. The search terms were strategically selected to encompass "epinephrine," "postpolypectomy bleeding," and "prophylaxis," along with their various permutations and combinations. This approach was aimed at capturing a wide range of relevant studies, thereby facilitating a comprehensive review of the available literature on the topic.

Inclusion and Exclusion Criteria

The inclusion criteria were focused on studies that examined the use of prophylactic epinephrine versus no prophylaxis in the context of postpolypectomy bleeding. Exclusion criteria played a crucial role in narrowing the review's scope. Studies were excluded if they involved mechanical prophylaxis in the control group, combined interventions, non-English language publications, or non-human studies. The rationale behind these exclusions was to ensure a focused comparison between chemical prophylaxis (epinephrine) and no intervention, thereby reducing confounding factors and enhancing the review's specificity and relevance.

Risk of Bias Assessment

The quality of the included studies was assessed and potential biases were evaluated using the Cochrane Risk of Bias assessment tool. This comprehensive tool evaluates several bias domains, including random sequence generation, allocation concealment, blinding of participants and personnel, blinding of outcome assessment, incomplete outcome data, selective reporting, and other potential biases. Each study was independently appraised by two reviewers to ensure an unbiased and objective assessment. This dual-review process was crucial in maintaining the integrity and validity of the review. A bias value of “high,” “unclear,” or “low” was given for each item. This rigorous evaluation was essential in ensuring that the findings of the review were robust, reliable, and could be confidently interpreted.

Data Extraction and Statistical Analysis

Data extraction was carried out using a predefined and standardized form, filled independently by two reviewers to guarantee accuracy and reduce the risk of data extraction errors. Extracted data included study characteristics, participant demographics, details of intervention and control conditions, outcomes measured, and the results obtained.

The statistical analysis was performed using a random-effects model, deemed appropriate for accommodating the expected variability among study results. Heterogeneity among studies was quantitatively assessed using the I^2^ statistic. This heterogeneity assessment was pivotal in understanding the variability in effect sizes across studies and in interpreting the results in the context of this variability.

Results

Search Outcomes and Selection Process

**Figure 1:**
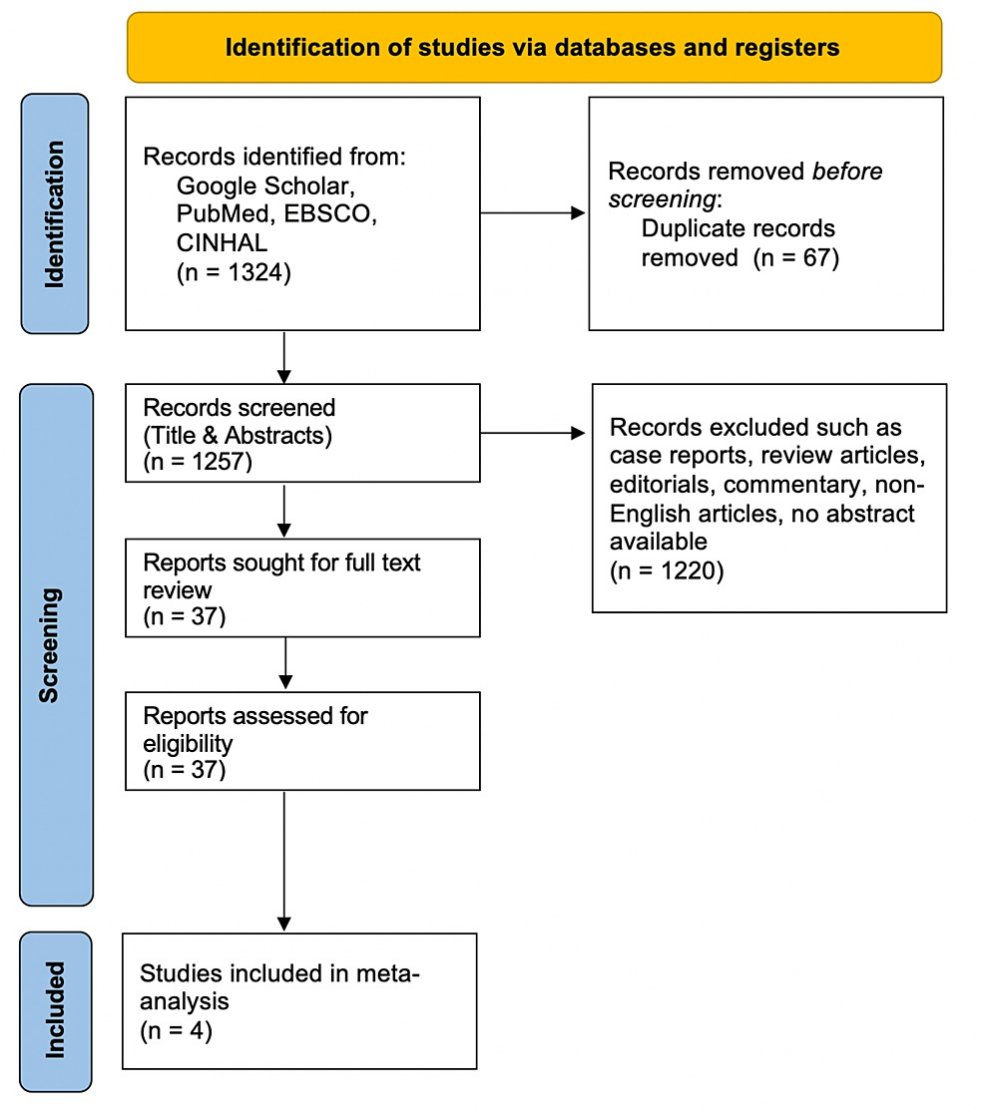
PRISMA chart PRISMA: Preferred Reporting Items for Systematic Reviews and Meta-Analyses

The initial comprehensive search yielded 1,324 relevant articles. Subsequent to duplicate removal (67 articles), a total of 1,257 articles underwent title and abstract screening. Application of the exclusion criteria further refined the pool to 37 articles for full-text review. Ultimately, only four studies met the inclusion criteria, specifically comparing bleeding rates in groups receiving prophylactic epinephrine versus no prophylaxis. The detailed selection process is elucidated in the Preferred Reporting Items for Systematic Reviews and Meta-Analyses (PRISMA) flow chart (Figure 1).

Study and Participant Characteristics

The characteristics of the included studies, along with participant demographics, are systematically summarized in Table 1. This table concisely presents key aspects such as study location, polyp size criteria, definitions of early and delayed bleeding, and group comparisons.

**Table 1:** Study and participant characteristics

Author name(s) and year	Country	Centers	Polyps size	Definition of early bleeding	Definition of Delayed bleeding	Comparisons group	Male in epinephrine group (%)	Male in control group (%)	Total polyps	Mean age of epinephrine group (years)	Mean age of control group (years)	Mean polyp size of epinephrine group	Mean polyp size of control group
Lee et al., 2009 [17]	South Korea	Multi-Center	>10mm	Hematochezia within 12 hr	24 hr to 30 days	Epinephrine group vs no prophylaxis	70.9	64.3	561	52±11.4	57±11.3	14.5±5.7 mm	15±6.8 mm
Hsieh et al., 2001 [18]	Taiwan	Single Center	Not available	During the procedure or within 24 hr	24 hr to 30 days	Epinephrine solution injection vs no prophylaxis	Not provided	Not provided	151	63(59-62)	65(62-68)	8mm (8-13)	8mm (8-11)
Dobrowolski et al., 2004 [14]	Poland	Single Center	≥10mm	Bleeding within 24 hrs	24 hr to 30 days	Epinephrine solution injection vs no prophylaxis	72	64	100	64±9.7	67±11.5	16±5.4 mm	16±5.9 mm
Di Giorgio et al., 2004 [13]	Italy	Single Center	>10mm	During the procedure or within 24 hr	24 hr to 30 days	Epinephrine solution injection vs no prophylaxis	54	53	488	64±9.2	62±9.0	22.2±5.9 mm	21.6±4.8 mm

Risk of Bias Evaluation

**Figure 2:**
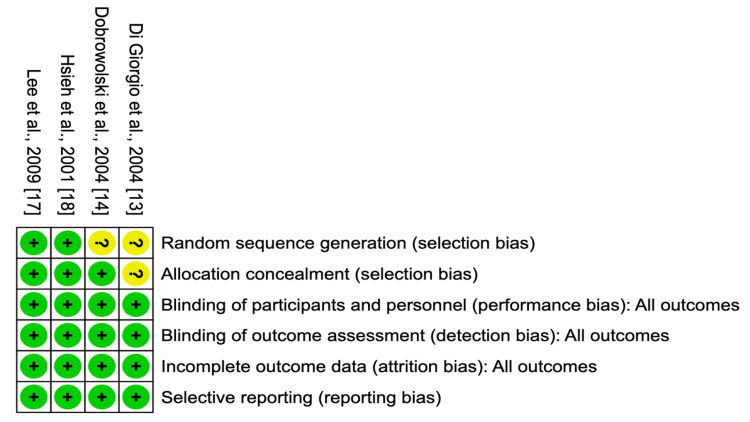
Cochrane risk of bias evaluation

The Cochrane Risk of Bias assessment tool was employed to evaluate the quality of the included studies. None of the studies exhibited a high risk of bias. However, two studies presented an unclear risk of selection bias, as depicted in Figure 2 [13,14].

Incidence and Comparative Analysis of Early Bleeding

**Figure 3:**
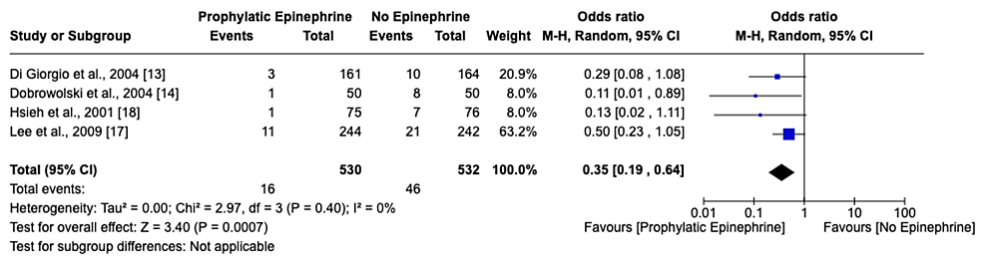
Forest plot and meta-analysis of early bleeding

In total, 1,062 patients were analyzed, with 530 in the epinephrine group and 532 in the control group. The incidence of early bleeding was significantly lower in the epinephrine group (3.02%) as compared to the no-prophylaxis group (8.65%). The calculated odds ratio of 0.35 (95% CI: 0.19-0.64) denotes a significantly reduced risk associated with epinephrine use. This finding is further corroborated by the low heterogeneity observed among the studies (I^2^=0%, p=0.41), as illustrated in Figure 3.

Incidence and Comparative Analysis of Delayed Bleeding

**Figure 4:**
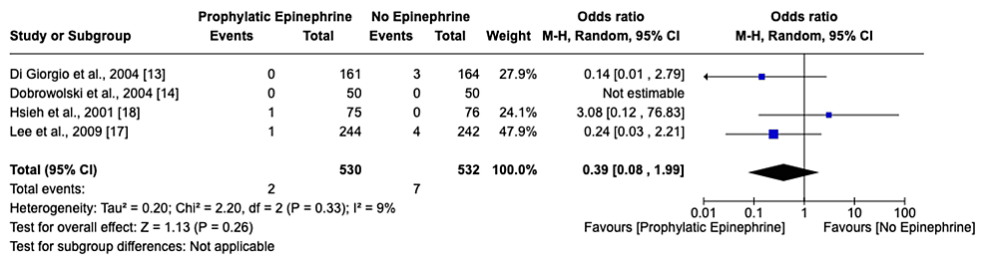
Forest plot and meta-analysis of delayed bleeding

A parallel analysis for delayed bleeding revealed lower incidences in both groups. Specifically, 0.38% of patients in the epinephrine group experienced delayed bleeding as compared to 1.32% in the control group. An odds ratio of 0.39 (95% CI: 0.08-1.99) suggests a reduced risk with epinephrine, although the broad confidence interval points to a need for further research for conclusive evidence. The heterogeneity among the studies was low (I^2^=9%, p=0.33), as shown in Figure 4.

Discussion

Our study makes a significant contribution to the current understanding of postpolypectomy bleeding, a pivotal concern in gastrointestinal endoscopic procedures. We observed a postpolypectomy bleeding rate of 6.68%, notably surpassing the previously reported prevalence of 0.75% to 5% [19,20]. In our cohort, epinephrine administration significantly reduced early or immediate postpolypectomy bleeding to 3%, in contrast to 8.64% in the no-prophylaxis group. This intervention did not show any effect on the incidence of delayed bleeding, which was 0.85%, exceeding the range of 0.14% to 0.74% noted in previous studies [19,21,22]. These findings offer nuanced insights into the differential impacts of prophylactic strategies during various postpolypectomy phases.

The elevated bleeding rate observed might reflect complex interplays involving patient characteristics and procedural nuances. The effectiveness of prophylactic epinephrine in mitigating early bleeding underscores its potential as a critical intervention in high-risk patients. However, its limited influence on delayed bleeding highlights the multifaceted nature of this complication, suggesting that delayed bleeding may be influenced by a broader array of factors, including patient comorbidities and extended healing period.

The study's strength lies in its focused assessment of postpolypectomy bleeding and the specific impact of prophylactic epinephrine. The comparative analysis between intervention and control groups strengthens the validity of our findings. However, the study is limited by its lack of extensive evaluation of other influential factors like patient comorbidities, variation in polypectomy techniques, and the use of antithrombotic medications. These omissions may have impacted the depth and breadth of our findings. The variability in data collection also poses limitations to the study's comprehensiveness.

Our study aligns with previous research in terms of the effectiveness of prophylactic epinephrine in reducing early postpolypectomy bleeding [23,24]. However, it diverges regarding delayed bleeding outcomes, where our findings indicate a lack of significant impact, contrasting with previous reports [19,21,22].

Our study's limitations include the absence of a detailed analysis of variables such as patient comorbidities, polypectomy techniques, and antithrombotic medication use potentially biases the results. Additionally, the methodological rigor varied, with some aspects of the study design and patient selection potentially impacting the findings' consistency and generalizability. The limitations in capturing rare or serious adverse events also pose significant constraints in comprehensively understanding the safety profile of prophylactic epinephrine. These factors collectively necessitate a cautious interpretation of our conclusions and underline the imperative for more detailed, methodologically robust future research in this domain.

## Conclusions

This systematic review and meta-analysis revealed that prophylactic epinephrine injection significantly reduces early postpolypectomy bleeding. However, its impact on delayed bleeding remains unclear, indicating the need for further research in this area. These findings highlight the importance of epinephrine as a prophylactic tool in colonoscopic polypectomy while also emphasizing the complexity of bleeding risks and the necessity for ongoing investigation.
